# The value of the preoperative Naples prognostic score in predicting prognosis in gallbladder cancer surgery patients

**DOI:** 10.1186/s12957-023-03198-0

**Published:** 2023-09-25

**Authors:** Jie Yang, Lin Lv, Fengqing Zhao, Xiaoping Mei, Hongkun Zhou, Feijie Yu

**Affiliations:** 1 Department of Hepatobiliary and Pancreatic Surgery, First Hospital of Jiaxing, Affiliated Hospital of Jiaxing University, Jiaxing, 314000 Zhejiang China; 2 Department of Health Management Center, First Hospital of Jiaxing, Affiliated Hospital of Jiaxing University, Jiaxing, 314000 Zhejiang China

**Keywords:** Gallbladder cancer, The naples prognostic score, Prognosis, Nomogram

## Abstract

**Purpose:**

The Naples prognostic score (NPS) is a comprehensive prognostic model that includes inflammatory and nutrition-related indicators and is increasingly used as a prognostic score for various malignant tumors. Given its predictive effect on prognosis in patients with gallbladder cancer, it is currently unclear. This study aimed to investigate the role of preoperative NPS in predicting prognosis in gallbladder cancer surgery patients.

**Patients and methods:**

A retrospective analysis was performed for 135 patients who underwent radical surgery for gallbladder cancer without preoperative treatment between March 2011 and January 2020. NPS was calculated by measuring the preoperative total cholesterol value, serum albumin value, neutrophil–lymphocyte ratio (NLR), and lymphocyte-monocyte ratio (LMR). They were then divided into 3 groups (groups 0, 1, and 2) based on NPS scores. Survival analysis was performed using the Kaplan–Meier method and log-rank test. Univariate and multivariate Cox proportional hazards models were used to identify independent prognostic factors. Plot time-dependent receiver operating characteristic (ROC) curves to compare the prognostic value of scoring systems. Finally, a nomogram model was developed with independent prognostic factors.

**Results:**

Multivariate analysis showed that NPS was an independent risk factor affecting OS (*HR* = 3.417, *p* < 0.05). The time-dependent ROC curve results showed that NPS had a better predictive value on survival prognosis than other indicators. The nomogram constructed according to independent factors such as NPS has a good predictive ability for OS.

**Conclusion:**

As a simple and reliable tool, the NPS has important predictive value in the survival prognosis of gallbladder cancer patients. The nomogram model constructed by NPS will help determine prognosis and make individualized treatment decisions.

## Introduction

Gallbladder cancer is one of the most common malignancies of the biliary system, with the fifth highest incidence among gastrointestinal tumors [1]. Radical surgical resection remains the most effective treatment. However, early diagnosis of gallbladder cancer is difficult because its early symptoms are often nonspecific, highly aggressive, and have a very poor prognosis, with a 5-year overall survival (OS) rate of less than 5% [2, 3].

Postoperative tumor pathologic features and TNM staging for gallbladder cancer can help assess prognosis and guide treatment strategies [4]. However, in clinical work, we have found that the prognosis of patients with the same stage may be significantly different, so in the current treatment and research of gallbladder cancer, it is necessary to explore other factors affecting the prognosis of gallbladder cancer.

Many studies have shown that the body’s inflammatory response and immune status play a very important role in the formation and development of malignant tumors. The body’s inflammatory response can promote angiogenesis and immunosuppression, have an impact on the tumor microenvironment, and ultimately promote tumor cell proliferation, invasion, and metastasis [5]. Inflammation-related scoring systems, such as systemic inflammation score (SIS) and neutrophil-lymphocyte ratio (NLR), have been shown to correlate with prognosis for esophageal, hepatocellocarcinoma, and lung cancer [6–8]. In addition, malignancy can cause malnutrition, resulting in immunosuppressive states and intolerance to chemotherapy [9]. Preoperative nutrition or immune status scoring systems, such as the prognostic nutrition index (PNI) and controlled nutritional status (CONUT), are also widely used to predict prognosis [10, 11].

The Naples prognostic score (NPS) is a new scoring system consisting of serum albumin, total cholesterol, NLR, and lymphocyte-monocytes ratio (LMR). Because it takes into account the body’s inflammatory response, nutrition, and immune status, it is more accurate in predicting survival than other prognostic factors [12]. The prognostic value of NPS in gallbladder cancer has been poorly reported. The objective of this study was to explore the relationship between the clinicopathological features and prognosis of NPS in gallbladder cancer patients. In addition, the new prognostic scoring system was compared with other scoring systems to assess its performance.

## Methods

### Patient cohort

A retrospective analysis was performed on 151 patients who underwent surgical resection in First Hospital of Jiaxing between March 2011 and January 2020. Inclusion criteria are as follows: (1) postoperative pathology was clearly defined as gallbladder cancer, (2) underwent radical surgical resection with a margin of R0, (3) patients with detailed and extractable medical data and laboratory results, and (4) age > 18 years old. Exclusion criteria are as follows: (1) patients receiving antitumor therapy before surgery, (2) patients who were not the first primary malignant tumor, and (3) there was a distant metastasis (M1). Among them, 6 cases of clinicopathological data were missing, and 7 cases of distant metastasis and 3 cases of R2 resection were excluded. In the end, 135 cases that met the criteria were included.

### Data collection

Results of peripheral blood tests within 1 week prior to surgery were collected: lymphocyte count, neutrophil count, monocytes count, serum albumin, alkaline phosphatase, and total cholesterol. Clinicopathological data are as follows: age, gender, T stage, lymph node metastasis, TNM stage, degree of tumor differentiation, and vascular and neural invasion.

In this study, OS was defined as the time from the end of surgery to death for any cause. The last follow-up was in August 2020. Survival data were extracted from outpatient records or telephone follow-ups during follow-up visits.

This study was conducted in compliance with the Declaration of Helsinki and was approved by the Research Ethics Committee of First Hospital of Jiaxing, batch number 2022-KY-597. We retrospectively used information about subjects’ previous clinical visits without direct contact with them and protected their privacy. The ethics committee of the First Hospital of Jiaxing has approved the request for waiver of informed consent for this study.

### NPS and other prognostic scoring systems

The NPS was calculated according to Galizia et al.’s method, with four parameters including serum albumin, total cholesterol, NLR, and LMR [12]. Albumin concentration < 4.0 mg/dl was scored as 1, while concentration ≥ 4.0 mg/dl was scored as 0. Total cholesterol ≤ 180 mg/dl was scored as 1, while total cholesterol > 180 mg/dl was scored as 0. *NLR* > 2.96 was scored as 1, while *NLR* ≤ 2.96 was scored as 0. *LMR* ≤ 4.44 was scored as 1, while *LMR* > 4.44 was scored as 0. The NPS was defined as the sum of the aforementioned scores. The patients were divided into three groups based on their NPS score: patients with a score of 0 were assigned to group 0, patients with a score of 1 or 2 were assigned to group 1, and patients with a score of 3 or 4 were assigned to group 2.

PNI was calculated as *PNI* = 10 × serum albumin (g/dL) + 5 × lymphocyte count (per nanoliter) [13]. Patients were divided into low and high PNI groups based on the optimal PNI cutoff of 57.45 determined by time-dependent receiver operating characteristic (ROC) curve. The CONUT score was based on blood albumin level, total lymphocyte count, and total cholesterol level [11]. The optimal CONUT cut-off value was calculated to be 2.5 points. For SIS, serum albumin level ≥ 40 g/L and LMR level ≥ 4.44 score 0; hypoalbuminemia (< 4.0 g/dl) or low LMR (< 4.44) score 1 [8].

### Statistical methods

In this study, we used either the χ^2^ test or the Fisher exact probability method to assess the relationship between NPS and the clinicopathological features of patients. The survival curve was plotted by the Kaplan–Meier method, and the log-rank test was carried out in parallel. Prognostic factors were evaluated by Cox regression model. To assess the discriminant ability of the prognostic scoring system, we compared the prognostic value of NPS, SIS, CONUT, and PNI using the predicted values of the time-dependent ROC curve and the area under curve (AUC). A higher AUC value indicates better predictive power. The *p*-value < 0.05 was statistically significant. SPSS 22.0 (IBM Corporation, Armonk, NY, USA) and R software (the R project for statistical computing, Vienna, Austria) were used for statistical analysis. The R-packet “timeROC” was used for time-dependent ROC curve analysis, and the nomogram was drawn using the R-packet “rms”.

## Results

### Patients’ characteristics

A total of 135 patients with gallbladder cancer were included in this study. Based on previous study, the age boundary was 60 years, and the male-to-female ratio was 1:2.3 [14, 15]. According to the eighth edition of the American Joint Committee on Cancer (AJCC) staging system, 96 cases (71.1%) were stages I or II, and 39 (28.9%) were stages III or IV. Among the included patients, 20 (14.8%) patients were classified into group 0 (NPS 0), 72 (53.3%) into group 1 (NPS 1 or 2), and 43 (31.9%) into group 2 (NPS 3 or 4). The analysis results of the relationship between NPS and clinicopathological features are shown in Table 1. NPS was closely related to the tumor differentiation (*p* = 0.001), and there was no significant correlation with age, gender, T stage, N stage, TNM stage, vascular invasion, and perineural invasion (all *p* > 0.05).

**Table 1:** Association of NPS and clinicopathological characteristics in patients with gallbladder cancer

		**Group**			
**Characteristic**	**Overall, *N* = 135**	**Group 0, *N* = 20**	**Group 1, *N* = 72**	**Group 2, *N* = 43**	***p*-value**
Age					0.446
< 60	34 (25.2%)	5 (25.0%)	21 (29.2%)	8 (18.6%)	
≥ 60	101 (74.8%)	15 (75.0%)	51 (70.8%)	35 (81.4%)	
Gender					0.060
Female	94 (69.6%)	18 (90.0%)	50 (69.4%)	26 (60.5%)	
Male	41 (30.4%)	2 (10.0%)	22 (30.6%)	17 (39.5%)	
T stage					0.778
T1/T2	117 (86.7%)	18 (90.0%)	63 (87.5%)	36 (83.7%)	
T3/T4	18 (13.3%)	2 (10.0%)	9 (12.5%)	7 (16.3%)	
N status					0.845
N0	105 (77.8%)	16 (80.0%)	57 (79.2%)	32 (74.4%)	
N1/N2	30 (22.2%)	4 (20.0%)	15 (20.8%)	11 (25.6%)	
TNM stage					0.781
I-II	96 (71.1%)	14 (70.0%)	53 (73.6%)	29 (67.4%)	
III-IV	39 (28.9%)	6 (30.0%)	19 (26.4%)	14 (32.6%)	
Differentiation					0.001
Well or moderately	68 (50.4%)	17 (85.0%)	36 (50.0%)	15 (34.9%)	
Poorly or undifferentiated	67 (49.6%)	3 (15.0%)	36 (50.0%)	28 (65.1%)	
Vascular invasion					0.580
Negative	108 (80.0%)	17 (85.0%)	59 (81.9%)	32 (74.4%)	
Positive	27 (20.0%)	3 (15.0%)	13 (18.1%)	11 (25.6%)	
Perineural invasion					0.429
Negative	89 (65.9%)	15 (75.0%)	44 (61.1%)	30 (69.8%)	
Positive	46 (34.1%)	5 (25.0%)	28 (38.9%)	13 (30.2%)	

### OS examined based on NPS

**Fig. 1:**
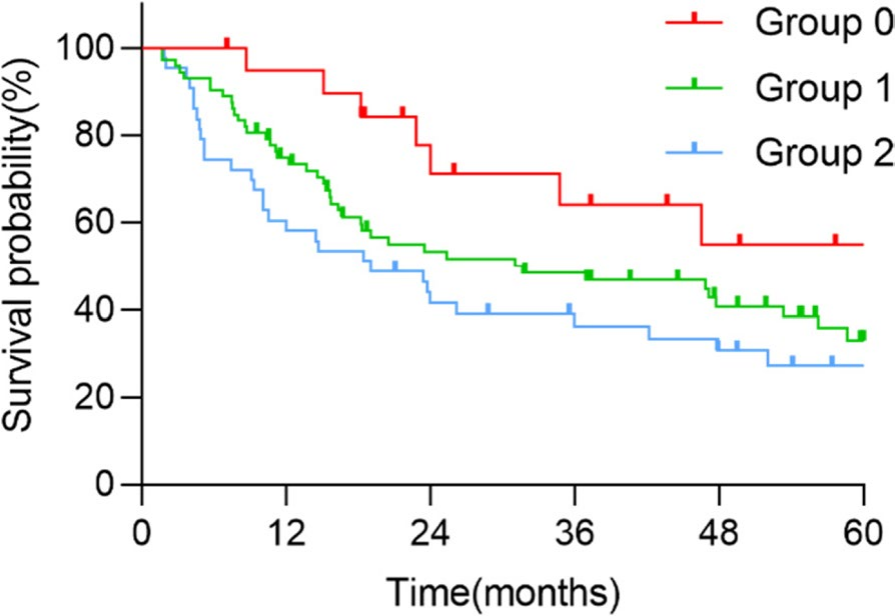
Kaplan-Meier survival analysis of OS according to NPS

As of the follow-up cut-off date, the median OS of the whole group was 31.0 months, and the survival rates of 1, 3, and 5 years were 72.4%, 46.9%, and 34.2%, respectively. The 1-, 3-, and 5-year OS rates of group 0 (0 point of NPS) patients were 94.7%, 64.1%, and 55.0%; group 1 (1 or 2 points of NPS) patients were 74.8%, 48.4%, and 33.0%, respectively; and group 2 (3 or 4 points of NPS) patients were 58.1%, 33.5%, and 27.3%, respectively, with statistically significant differences (χ^2^ = 6.029, *p* < 0.05). The Kaplan–Meier survival curve is shown in Fig. 1, and the OS is significantly shortened as the NPS group progressively increases. NPS group was significantly associated with OS (log rank, *p* = 0.014).

### Univariate and multivariate analyses of prognostic factors in gallbladder cancer

Univariate survival analysis of Table 2 showed that NPS, T stage, N stage, TNM stage, degree of differentiation, vascular invasion, and perineural invasion were closely related to OS in gallbladder cancer patients (all *p* < 0.05).

**Table 2:** Univariate analysis of clinicopathologic variables in relation to OS in patients with gallbladder cancer

	**Univariate analysis**		
**Characteristic**	**HR**	**95% *CI***	***p*-value**
Age (< 60, ≥ 60)	1.713	0.990-2.967	0.055
Gender (female, male)	1.008	0.628-1.619	0.972
T stage (T1/T2, T3/T4)	3.159	1.802-5.541	< 0.001
N status (N0, N1/N2)	2.766	1.711-4.472	< 0.001
TNM stage (I-II, III-IV)	3.061	1.945-4.817	< 0.001
Differentiation (well or moderately, poorly or undifferentiated)	3.203	2.009-5.108	< 0.001
Vascular invasion (positive, negative)	5.306	3.172-8.875	< 0.001
Perineural invasion (positive, negative)	4.967	3.122-7.904	< 0.001
NPS			
0			
1	1.983	0.891-4.417	0.094
2	2.754	1.212-6.258	0.016

*Abbreviations: NPS* naples prognostic score

**Fig. 2:**
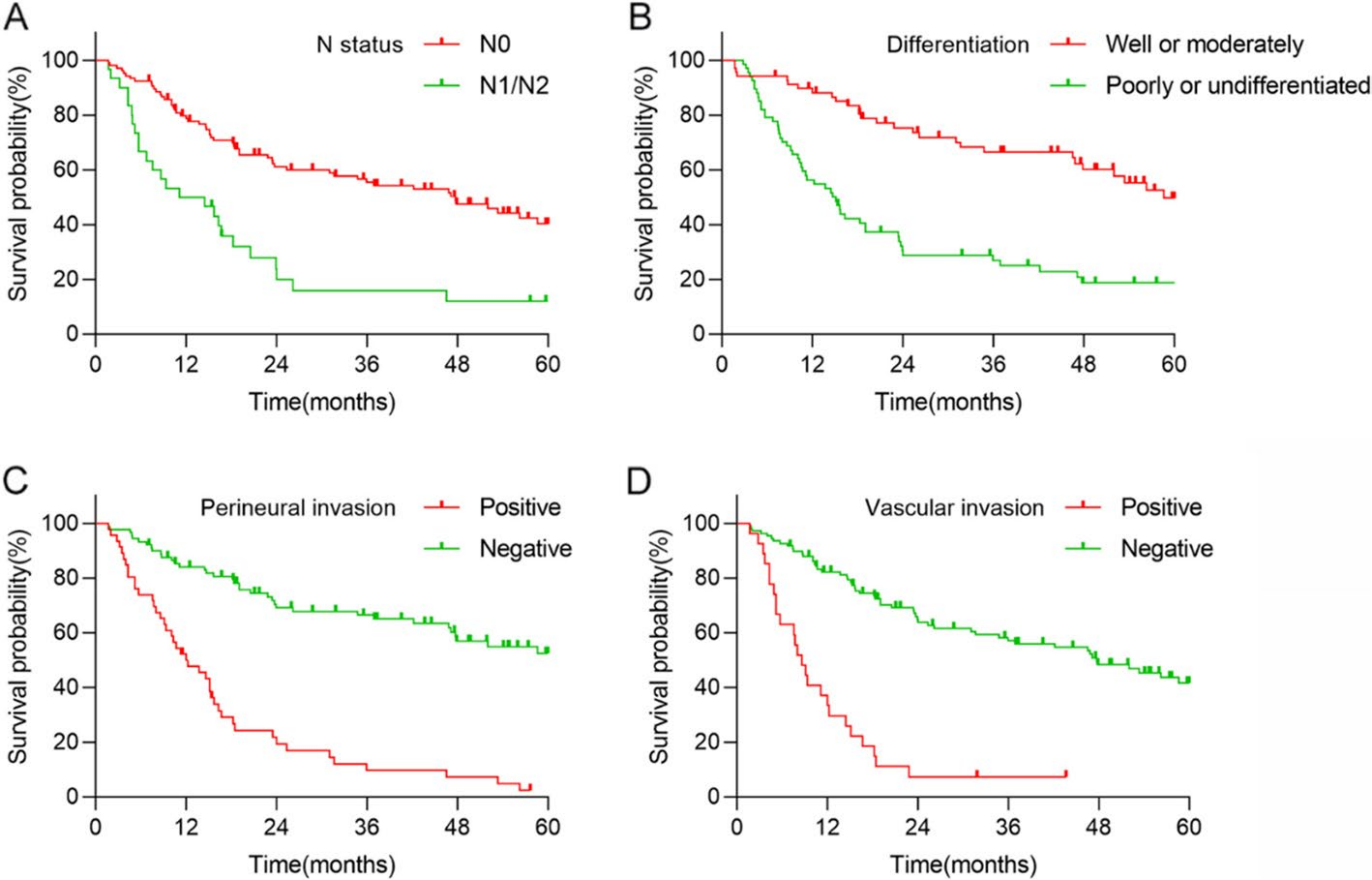
Kaplan-Meier survival analysis of OS according to N status (**A**), differentiation (**B**), perineural invasion (**C**), and vascular invasion (**D**)

According to Cox multivariate analysis, NPS is an independent risk factor affecting the postoperative prognosis of gallbladder cancer patients. The degree of differentiation (*HR* = 2.276, *p* = 0.001), vascular invasion (*HR* = 3.184, *p* < 0.001), perineural invasion (*HR* = 3.929, *p* < 0.001), and N stage (*HR* = 1.770, *p* < 0.05) showed significant correlations with OS in gallbladder cancer patients (Fig. 2, Table 3).

**Table 3:** Multivariate analysis of clinicopathologic variables in relation to OS in patients with gallbladder cancer

	**Multivariable analysis**		
**Characteristic**	**HR**	**95% *CI***	***p*-value**
T stage (T1/T2, T3/T4)			0.635
N status (N0, N1/N2)	1.770	1.058-2.961	0.030
TNM stage (I-II, III-IV)			0.589
Differentiation (well or moderately, poorly or undifferentiated)	2.276	1.390-3.727	0.001
Vascular invasion (positive, negative)	3.184	1.784-5.682	< 0.001
Perineural invasion (positive, negative)	3.929	2.325-6.639	< 0.001
NPS			
0			
1	2.531	1.085-5.904	0.032
2	3.417	1.419-8.227	0.006

*Abbreviations:* *NPS*, naples prognostic score

### Discriminatory ability of the prognostic scoring systems

**Fig. 3:**
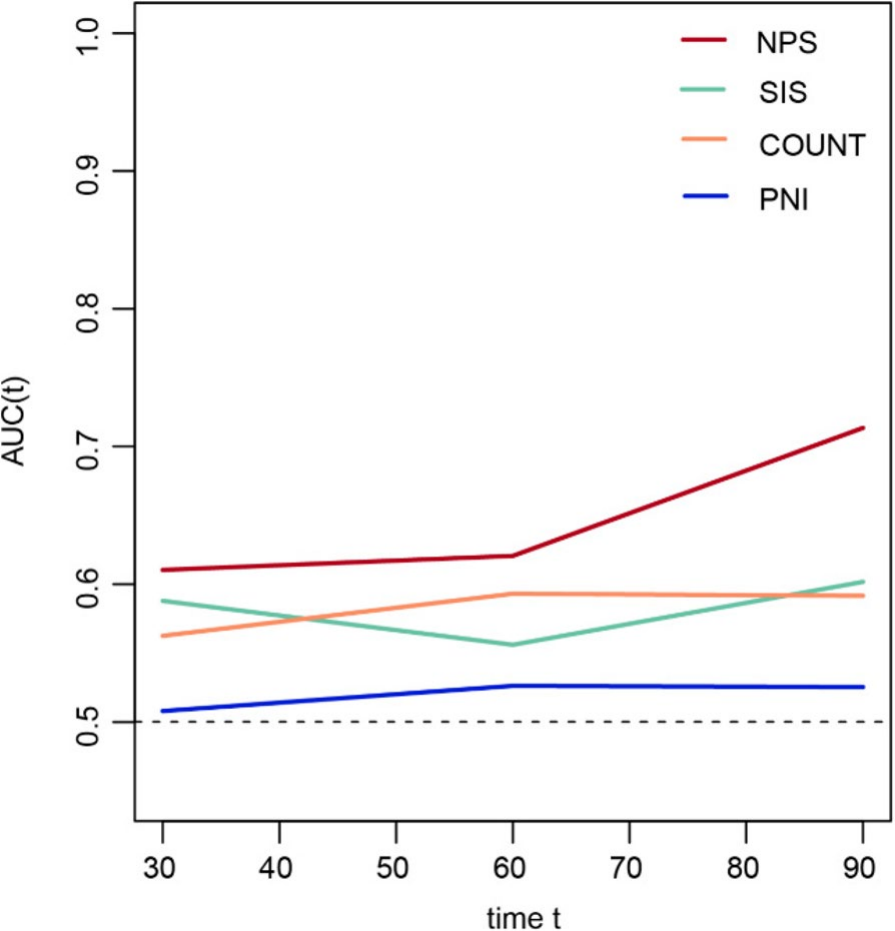
The time-dependent ROC curve analyses of prediction models for OS. The *X*-axis symbolizes the follow-up time, and the *Y*-axis represents estimated AUC for survival at specific time of interest

The prognostic value of NPS was compared to prognostic factors such as PNI, CONUT, and SIS. A time-dependent ROC curve was generated for each prognostic scoring system, and AUC values were calculated at different time points. The results of time-dependent ROC curve analysis showed that NPS showed better prognostic prediction performance than PNI, CONUT, and SIS at most time points (Fig. 3).

### Establishment and evaluation of nomogram model

**Fig. 4:**
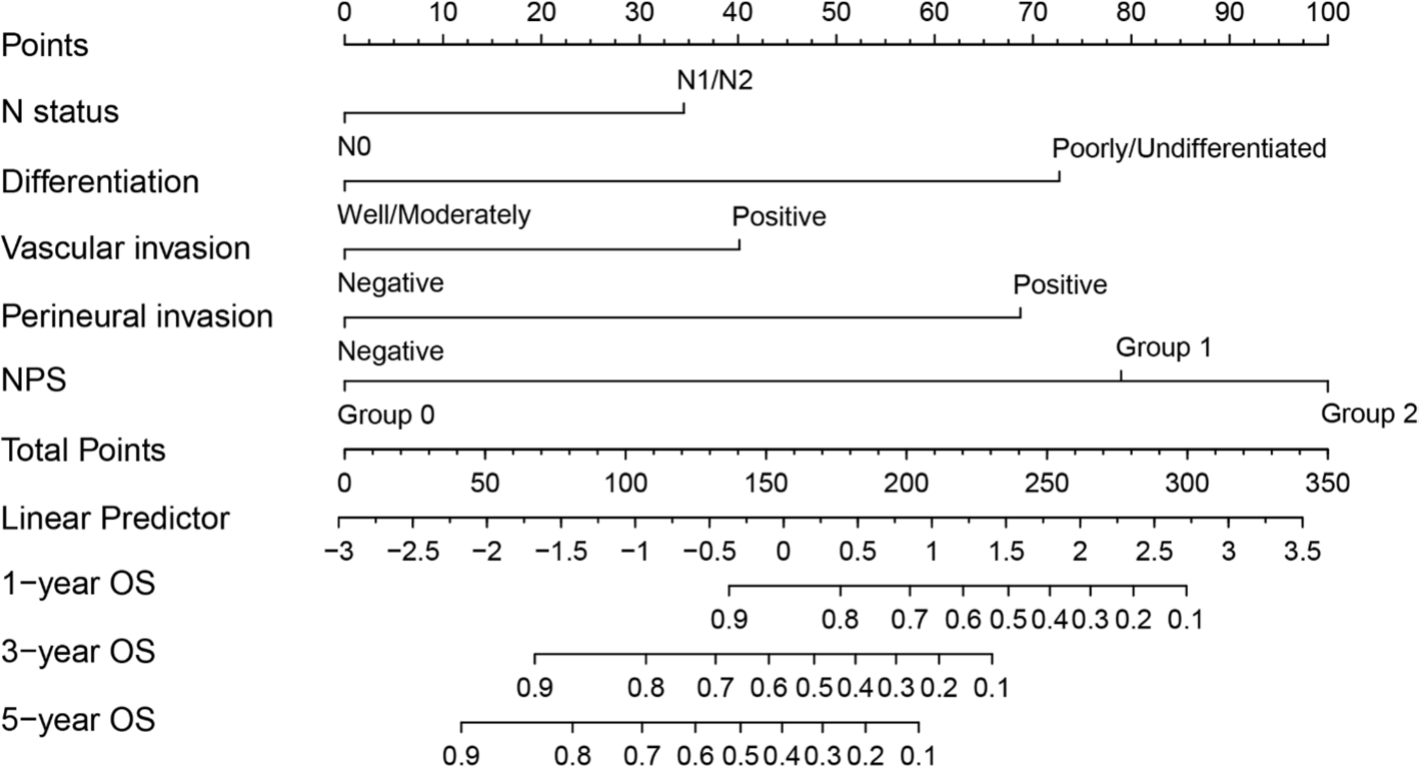
The nomogram based on NPS and clinical prognostic factors to predict the probabilities of 1-, 3-, and 5-year OS

**Fig. 5:**
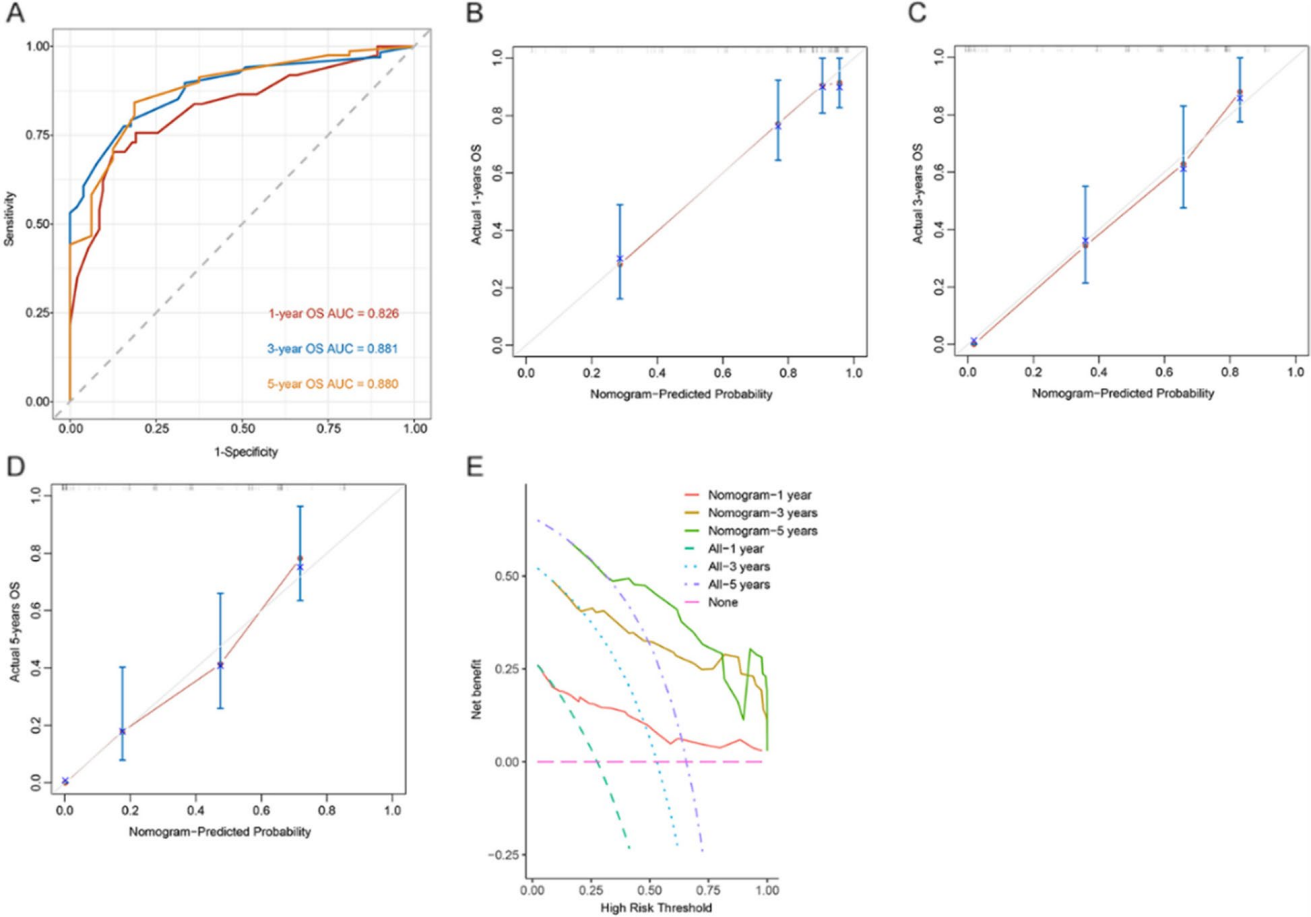
ROC curve of a predictive model that predicts 1-, 3-, and 5-year survival (**A**). One-, 3-, and 5-year calibration curve (**B**, **C**, **D**). One-, 3-, and 5-year clinical decision curve (**E**)

Based on the results of univariate and multivariate COX regression analysis, independent prognostic factors were integrated into the construction of nomogram models to predict OS at 1, 3, and 5 years (Fig. 4). The ROC curve results showed that the AUC values for 1 year, 3 years, and 5 years are 0.826, 0.881, and 0.880, respectively (Fig. 5A). In addition, the calibration curves of 1-year, 3-year, and 5-year survival probabilities showed that the predicted survival rate of the nomogram model was in good agreement with the actual observed values (Fig. 5B–D). The decision curves of 1, 3, and 5 years showed high net benefit values, indicating that the model has good clinical application value (Fig. 5E).

## Discussion

There is growing evidence that systemic inflammatory states and local immune responses play a role in tumor progression and survival in cancer patients [16]. Tissue damage caused by chronic inflammation produces local anti-inflammatory cytokines that promote tumor cell proliferation [17]. Interactions between immune cells and tumor cells induced tumor local immune tolerance and suppression of antitumor T-cell responses [18]. NLR, MLR, SIS, and other indicators to quantify systemic inflammatory response have been shown to be significantly associated with prognosis in various types of cancer [19–21].

Malnourished cancer patients are more likely to suffer from poor treatment tolerance, longer hospital stays, and reduced quality of life. Historically, malnutrition has been largely attributed to tumor depletion, but recent studies have suggested that inflammatory responses and immunosuppression are associated with malnutrition [22]. Serum albumin and total cholesterol as the main nutritional indicators not only reflect the overall nutritional status of the patient but also closely related to the prognosis of various types of cancer [11, 23]. Thus, early nutritional support for precachexia cancer patients can improve patient fitness, increase tolerability to antitumor therapy, and improve patient outcomes [24].

Gallbladder cancer is an aggressive tumor with a high risk of recurrence and death. R0 resection, N stage, tumor histologic grade, and nerve and vascular infiltration are independent prognostic factors affecting OS [25]. However, it is still difficult to accurately judge the prognosis of gallbladder cancer. In recent years, inflammatory, nutrition-related biomarkers have been found to be valuable in predicting the prognosis of gallbladder cancer patients. Studies have shown that elevated NLR is associated with a poorer prognosis after radical resection of gallbladder cancer [26]. PNI serves as an indicator of nutrition, immunity, and risk factors for OS of gallbladder cancer [27]. The limitations of these biomarkers lie in the individual differences in the included cases and the different laboratory standards, which makes it impossible to establish a uniform threshold of standards and limits clinical application.

NPS is a nutritional inflammation assessment index established by the researchers, which was originally used to predict postoperative survival in colorectal cancer patients after surgical treatment. The four indicators that make up NPS are obtained by routine peripheral blood laboratory testing before surgery. NPS combines NLR, lymphocytes and albumin, and other biomarkers to link the effects of cancer, nutrition, inflammation, and immunity and has a powerful prognosis prediction function. In this study, the prognosis of 135 gallbladder cancer patients who underwent radical surgery found that preoperative NPS was independent influencing factor of OS. The higher the NPS, the worse the patient’s prognosis. We compared the predictions of different prognostic scores by time-dependent ROC curve analysis. The results showed that NPS had the highest AUC value compared with PNI, CONUT, and SIS, and the prognosis prediction effect was the best.

With the advantages of vision and accuracy, the clinical prediction model of nomogram is becoming more and more widely used in tumor research. Based on the results of univariate and multivariate COX regression analysis, a nomogram predicting the prognosis of gallbladder cancer patients after radical surgery was constructed. The nomogram excels in predictive performance, with ROC curves showing AUC values of 0.826, 0.881, and 0.880 for 1, 3, and 5 years, respectively. The calibration curve of the model visually reflected the good agreement between the predicted values and the actual observations. Therefore, this nomogram provided a convenient and individualized tool for OS prediction in gallbladder cancer patients.

This study has several limitations. First, as a retrospective cohort study, this study inevitably had potential selection bias. Second, the present study, which lacked external validation, was a single-center cohort study, and the moderate sample size might account for the attenuation of demonstrative power. Further prospective and multi-institutional studies are needed to confirm the findings of this study. And only preoperative serum indexes were paid attention to, and the dynamic changes of NPS during the entire treatment process were not analyzed.

## Conclusion

As a simple and convenient scoring system, NPS can be used to predict the efficacy and prognosis of gallbladder cancer patients treated with radical surgery. A new NPS-based nomogram prediction model is established and validated, which can be visualized and intuitively applied to clinical prognosis judgment and decision-making guidance.

## Data Availability

Raw data were generated at the Department of Hepatobiliary and Pancreatic Surgery, First Hospital of Jiaxing. The datasets used and/or analyzed during the current study are available from the corresponding author on reasonable request.

## Abbreviations

NPS: Naples prognostic score
SIS: Systemic inflammation score
CONUT: Controlling nutritional status
PNI: Prognostic nutrition index
NLR: Neutrophil-lymphocyte ratio
LMR: Lymphocyte-monocyte ratio
AUC: Area under the curve
OS: Overall survival
ROC: Receiver operating characteristic

## References

[1] Oh TG, Chung MJ, Bang S, Park SW, Chung JB, Song SY (2013). Comparison of the sixth and seventh editions of the AJCC TNM classification for gallbladder cancer. J Gastrointest Surg.

[2] Li H, Yuan SL, Han ZZ, Huang J, Cui L, Jiang CQ (2017). Prognostic significance of the tumor-stroma ratio in gallbladder cancer. Neoplasma.

[3] Wang FT, Li XP, Pan MS, Hassan M, Sun W, Fan YZ (2021). Identification of the prognostic value of elevated ANGPTL4 expression in gallbladder cancer-associated fibroblasts. Cancer Med.

[4] Mishra PK, Saluja SS, Prithiviraj N, Varshney V, Goel N, Patil N (2017). Predictors of curative resection and long term survival of gallbladder cancer - a retrospective analysis. Am J Surg.

[5] Dumitru CA, Lang S, Brandau S (2013). Modulation of neutrophil granulocytes in the tumor microenvironment: mechanisms and consequences for tumor progression. Semin Cancer Biol.

[6] Diem S, Schmid S, Krapf M, Flatz L, Born D, Jochum W (2017). Neutrophil-to-lymphocyte ratio (NLR) and platelet-to-lymphocyte ratio (PLR) as prognostic markers in patients with non-small cell lung cancer (NSCLC) treated with nivolumab. Lung Cancer (Amsterdam, Netherlands).

[7] Fu X, Li T, Dai Y, Li J (2019). Preoperative systemic inflammation score (SIS) is superior to neutrophil to lymphocyte ratio (NLR) as a predicting indicator in patients with esophageal squamous cell carcinoma. BMC Cancer.

[8] Inokuchi S, Itoh S, Yoshizumi T, Morinaga A, Toshima T, Takeishi K (2021). Prognostic significance of systemic inflammation score in patients who undergo hepatic resection for hepatocellular carcinoma. Langenbecks Arch Surg.

[9] Klute KA, Brouwer J, Jhawer M, Sachs H, Gangadin A, Ocean A, et al. Chemotherapy dose intensity predicted by baseline nutrition assessment in gastrointestinal malignancies: a multicentre analysis. Eur J Cancer (Oxford, England : 1990). 2016;63:189–200. 10.1016/j.ejca.2016.05.011 27362999

[10] Itoh S, Tsujita E, Fukuzawa K, Sugimachi K, Iguchi T, Ninomiya M, et al. Prognostic significance of preoperative PNI and CA19–9 for pancreatic ductal adenocarcinoma: a multi-institutional retrospective study. Pancreatology. 2021;21(7):1356–63. 10.1016/j.pan.2021.08.003 34426076

[11] Kuroda D, Sawayama H, Kurashige J, Iwatsuki M, Eto T, Tokunaga R (2018). Controlling nutritional status (CONUT) score is a prognostic marker for gastric cancer patients after curative resection. Gastric Cancer.

[12] Galizia G, Lieto E, Auricchio A, Cardella F, Mabilia A, Podzemny V (2017). Naples prognostic score, based on nutritional and inflammatory status, is an independent predictor of long-term outcome in patients undergoing surgery for colorectal cancer. Dis Colon Rectum.

[13] Wang Z, Zhao L, He S (2021). Prognostic nutritional index and the risk of mortality in patients with hypertrophic cardiomyopathy. Int J Cardiol.

[14] Zhang W, Hong HJ, Chen YL (2018). Establishment of a gallbladder cancer-specific survival model to predict prognosis in non-metastatic gallbladder cancer patients after surgical resection. Dig Dis Sci.

[15] Bao Y, Yang J, Duan Y, Chen Y, Chen W, Sun D (2021). The C-reactive protein to albumin ratio is an excellent prognostic predictor for gallbladder cancer. Biosci Trends.

[16] Dupré A, Malik HZ (2018). Inflammation and cancer: what a surgical oncologist should know. Eur J Surg Oncol.

[17] Karin M, Greten FR (2005). NF-kappaB: linking inflammation and immunity to cancer development and progression. Nat Rev Immunol.

[18] Elinav E, Nowarski R, Thaiss CA, Hu B, Jin C, Flavell RA (2013). Inflammation-induced cancer: crosstalk between tumours, immune cells and microorganisms. Nat Rev Cancer.

[19] Templeton AJ, McNamara MG, Šeruga B, Vera-Badillo FE, Aneja P, Ocaña A, et al. Prognostic role of neutrophil-to-lymphocyte ratio in solid tumors: a systematic review and meta-analysis. J Nat’l Cancer Inst. 2014;106(6):dju124. 10.1093/jnci/dju124 24875653

[20] Ethier JL, Desautels D, Templeton A, Shah PS, Amir E (2017). Prognostic role of neutrophil-to-lymphocyte ratio in breast cancer: a systematic review and meta-analysis. Breast Cancer Res.

[21] Kang J, Chang Y, Ahn J, Oh S, Koo DH, Lee YG (2019). Neutrophil-to-lymphocyte ratio and risk of lung cancer mortality in a low-risk population: a cohort study. Int J Cancer.

[22] Alwarawrah Y, Kiernan K, MacIver NJ (2018). Changes in nutritional status impact immune cell metabolism and function. Front Immunol.

[23] Königsbrügge O, Posch F, Riedl J, Reitter EM, Zielinski C, Pabinger I (2016). Association between decreased serum albumin with risk of venous thromboembolism and mortality in cancer patients. Oncologist.

[24] Fearon KC, Voss AC, Hustead DS (2006). Definition of cancer cachexia: effect of weight loss, reduced food intake, and systemic inflammation on functional status and prognosis. Am J Clin Nutr.

[25] Choi SB, Han HJ, Kim CY, Kim WB, Song TJ, Suh SO (2010). Surgical outcomes and prognostic factors for T2 gallbladder cancer following surgical resection. J Gastrointest Surg.

[26] Beal EW, Wei L, Ethun CG, Black SM, Dillhoff M, Salem A (2016). Elevated NLR in gallbladder cancer and cholangiocarcinoma - making bad cancers even worse: results from the US Extrahepatic Biliary Malignancy Consortium. HPB (Oxford).

[27] Cao P, Hong H, Yu Z, Chen G, Qi S (2022). A novel clinically prognostic stratification based on prognostic nutritional index status and histological grade in patients with gallbladder cancer after radical surgery. Front Nutr.

